# E2F transcription factor 1 (E2F1) promotes the transforming growth factor TGF-β1 induced human cardiac fibroblasts differentiation through promoting the transcription of CCNE2 gene

**DOI:** 10.1080/21655979.2021.1972194

**Published:** 2021-09-14

**Authors:** Rongheng Liao, Bo Xie, Jun Cui, Zhen Qi, Song Xue, Yongyi Wang

**Affiliations:** a Department of Cardiovascular Surgery, Renji Hospital, School of Medicine, Shanghai Jiao Tong University, Shanghai, China; b Department of Cardiovascular Surgery, Xijing Hospital, The Fourth Military Medical University, Xi’an, China

**Keywords:** Cardiac fibrosis, E2F1, CCNE2, human cardiac fibroblasts, myocardial infarction

## Abstract

The differentiation of cardiac fibroblast to myofibroblast is the key process of cardiac fibrosis. In the study, we aimed to determine the function of E2F Transcription Factor 1 (E2F1) in human cardiac fibroblasts (HCFs) differentiation, search for its downstream genes and elucidate the function of them in HCFs differentiation. As a result, we found that E2F1 was up-regulated in TGF-β1-induced HCFs differentiation. Silencing the expression of E2F1 by siRNA in HCFs, we found that the expression of differentiation-related genes (Collagen-1, α-Smooth muscle actin, and Fibronectin-1) was significantly suppressed, combining with proliferation and migration assay, we determined that HCFs differentiation was decreased. Luciferase report assay and immunoprecipitation proved that the oncogene CCNE2 was a direct target gene of E2F1, overexpression of CCNE2 was found in differentiated HCFs, silencing the expression of CCNE2 by siRNA decreased HCFs differentiation. Our research suggested that E2F1 and its downstream target gene CCNE2 play a vital role in TGF-β1-induced HCFs differentiation, thus E2F1 and CCNE2 may be a potential therapeutic target for cardiac fibrosis.

## Introduction

Myocardial infarction (MI) remains to be the leading cause of mortality and morbidity in humans, after MI, cardiac fibroblasts are activated to avoid cardiac rapture, but over-activation of cardiac fibroblasts will lead to cardiac fibrosis. Cardiac fibrosis is characterized by cardiac fibroblast-to-myofibroblast differentiation and extracellular matrix (ECM) deposition [1], is wildly involved in the cardiac remodeling and heart failure at the end stage of MI [2]. Despite the mechanism of this procedure has not been revealed completely yet, transforming growth factor-β1 (TGF-β1), a multifunctional growth factor, is now recognized as one of the most important stimuli contributed to this process [3,4]. Under the stimulation of TGF-β1, human cardiac fibroblasts (HCFs) are differentiated into myofibroblasts and secrete ECM [5], this phenotype change can be measured by the expression of α-smooth muscle actin (α-SMA) [6] and another ECM proteins like collagen type I (approximately 85%), collagen type III (approximately 11%) [7], and fibronectin-1 (FN1) [8]. Besides, HCF differentiation is characterized by enhanced proliferation and migration abilities. When MI occurs, fibroblast-to-myofibroblast differentiation is vital to maintain the structure of heart, but excessive activation of this process will lead to myocardial dysfunction and heart failure at the terminal stage of MI [9].

E2F transcription factor 1 (E2F1) is one of the E2F transcription factors family. Recent research shows that E2F family of transcription factors are the key regulators of numerous genes involved in DNA damage repair, cell apoptosis, and cell cycle progression [10,11]. The family exhibits both gene activation and repression activities. E2F1-2 and E2F3a are classified as gene expression activators whereas E2F3b, E2F4-8 are classified as gene expression inhibitors [12–14]. As the most characteristic member of the E2F family, E2F1 plays an important role in cell death, cell proliferation, and many pathological processes like liver fibrosis [15,16]. But its role in cardiac fibrosis and its downstream mechanism remain unclear.

In this research, we aimed to determine the function of E2F1 in HCFs differentiation, search for its downstream genes and elucidate the function of them in HCFs differentiation. In our hypothesis, we assumed that E2F1 participated in TGF-β1-induced HCFs differentiation by promoting the transcription activity of its downstream gene CCNE2. Our data showed that in HCFs, E2F1 promoted the fibroblast-to-myofibroblast differentiation. What’s more, we found that E2F1 directly promoted the transcriptional activity of its downstream target gene CCNE2. Suppression of CCNE2 attenuated the differentiation of myofibroblast after TGF-β1 treatment. To our knowledge, this finding was also the first time to illustrate E2F1 and CCNE2 genes in regulating HCFs differentiation.

## Methods

### Cell culture and transfection

Following our previous routines [17], human cardiac fibroblasts (HCFs) were obtained from Sciencell Research Lab (6300, USA). HCFs were cultured in DMEM/F-12 with 5% FBS (Gibco) 2 mM glutamine and 1% penicillin/streptomycin (Gibco) on condition of 37°C with 5% CO_2_. To avoid the impact of biomechanical input, the culture plates used were pre-coated with gelatin. The 3 to 6 passages of cultured cells were used in our experiments. Before transfection, HCFs were starved in DMEM/F12 without serum for 6 h and incubated with 10 ng/ml TGF-β1 (with DMEM/F12 and serum. Sino biology, Beijing, China). In our research, E2F1 siRNA (100 pmol/2 mL) and pcDNA3.1 (4 μg/2 mL), and CCNE2 siRNA (100 pmol/2 mL) were transiently transfected by Lipofectamine 3000 for 8 h. The E2F1 siRNA (100 nM), E2F1 pcDNA3.1 (100 nM), NC siRNA (100 nM), NC pcDNA3.1 (100 nM), CCNE2 siRNA (50 nM), and siRNA negative control (50 nM) were purchased from Sangon (Shanghai, China). Following the guidance of manufacturer’s instruction, all these procedures were performed.

### Quantitative realtime RT-PCR

Total RNAs were extracted by EZ-press RNA Purification Kit (EZ-bioscience) and reverse transcribed by HiScript III RT SuperMix for qPCR (+gDNA wiper) cDNA Synthesis Kit (Vazyme, Nanjing, China) by following manufacturer’s instruction. Quantitative realtime RT-PCR was performed by ChamQ SYBR GREEN qPCR Master Mix (Vazyme, Nanjing, China). The sequences of primers in this experiment are listed below: α-SMA (Forward: AAAAGACAGCTACGTGGGTGA, Reverse: GCCATGTTCTATCGGGTACTTC), COL-1A1 (Forward: GAGGGCCAAGACGAAGACATC, Reverse: CAGATCACGTCATCGCACAAC), FN1 (Forward: CGGTGGCTGTCAGTCAAAG, Reverse: AAACCTCGGCTTCCTCCATAA), E2F1 (Forward: ACGCTATGAGACCTCACTGAA, Reverse: TCCTGGGTCAACCCCTCAAG), E2F2 (Forward: CGTCCCTGAGTTCCCAACC, Reverse: GCGAAGTGTCATACCGAGTCTT), E2F3 (Forward: AGAAAGCGGTCATCAGTACCT, Reverse: TGGACTTCGTAGTGCAGCTCT), CCNE2 (Forward: TCAAGACGAAGTAGCCGTTTAC, Reverse: TGACATCCTGGGTAGTTTTCCTC), GAPDH (used as the reference gene, Forward: GGAGCGAGATCCCTCCAAAAT, Reverse: GGCTGTTGTCATACTTCTCATGG).

### Western blot analysis

RIPA (Beyotime, China) was used for harvesting cell lysates, and BCA protein assay kit (Beyotime, USA) was used for determining the concentration of protein samples. Same amounts of proteins were separated by SDS-PAGE gels (10%) and transferred to PVDF membranes. After 2 h of blocking in 5% Bovine Serum Albumin (BSA, Thermofisher, USA), PVDF membranes were incubated in primary antibodies under the temperature of room (24°C) for 2 h. After that, PVDF membranes were washed 3 times in 15 min with TBST and incubated in horseradish peroxidase-conjugated secondary antibody (Beyotime, China) for 1 hunder 24°C. Finally, an enhanced chemiluminescence (ECL, Vazyme, Nanjing, China) was used for imaging western blots. Image data were analyzed by ImageJ software (NIH, MD, USA). The antibodies used in this experiment are listed as below Anti-FN-1 (Rabbit, 1:3000, Abcam, MA, USA), anti-Col-1a1 (Rabbit, 1:1000, Abclonal, Wuhan, China), anti-α-SMA (Rabbit, 1:1000, Abcam, MA, USA), anti-E2F1 (Rabbit, 1:1000, Abclonal, Wuhan, China), anti-CCNE2 (Rabbit, 1:1000, Abclonal, Wuhan, China), anti-GAPDH (Rabbit, 1:3000, CST, MA, USA) for western-blot.

### Cell proliferation assay (EDU assay)

12-well plates with microscope slides were used for culturing HCFs, after 24 h of incubating, EDU Alexa Flour 555 imaging kit (Ribobio, Guangzhou, China) was used for detecting the proliferation of HCFs. All procedures were performed by following the manufacturer’s instructions. HCFs were incubated for 2 h after adding 1 μL EDU solution into each well. After incubating, HCFs were fixed in 75% ethyl alcohol on condition of −20°C overnight, then, fixed cells were penetrated by 0.3% PBST for 30 min, after washing 3 times in 0.1% TBST for 15 min. HCFs were incubated in EDU click solution (with Alexa Flour 564) away from light for 30 min. Images were captured by fluorescence microscope (magnification: ×400; Olympus, Japan). The proliferation of HCFs were calculated by counting the percentage of EDU positive cells (red fluorescence) to DAPI stained cells (blue fluorescence).

### Wound healing assay

6-well plates were used for culturing HCFs to 90% confluence and starved the HCFs into serum-free medium for 6 h to avoid the effect of serum on cell migration. After starving, 6-well plates were scratched with a 200 μl pipette tip by following the guidance of scratch ruler. PBS was used for washing plates, after three times of washing, the cells were incubated in different treatment groups (listed in legends of figures). Images were captured at 0 h and 24 h after scratching by an inverted light microscope (magnification: ×100, Olympus, Japan) and the wound closure (%) was accessed.

### Transwell migration assay

Transwell chamber (6-well insert, Corning, New York, USA) was used for culturing HCFs. To evaluate the migration ability of HCFs in transwell migration assay, 1 × 10^5^ cells were resuspended in 500 μl of DMEM/F12 serum-free medium in the upper chamber after different treatments (listed in legends of figures), 500 μl of DMEM/F12 medium supplemented with 25% FBS was added in the lower chamber. After 24 h of incubating, the migrated cells were fixed in 4% formaldehyde and penetrated by 0.3% PBST for 30 min, after washing 3 times in 0.1% TBST for 15 min, HCFs were stained with crystal violet (Beyotime, China). Under a light microscope (magnification: ×100, Olympus, Japan), five fields were randomly selected, and the average number of migrated HCFs was calculated to evaluate the ability of migration.

### Luciferase reporter assay

Two pmirGLO dual luciferase vectors (Shanghai Bioegene Co., Ltd.) were designed to carry CCNE2 5ʹUTR sequences of wild type and mutant E2F1 binding sites, respectively. Then, vectors and pcDNA3.1s (to overexpress E2F1) were transfected into HCFs by Lipofectamine 3000 (invitrogen). After 24 h, luciferase activity was detected by a dual-luciferase assay kit (vazyme, Nanjing, China) under the guidance of manufacturer’s instructions.

### Immunoprecipitation

6-well plates were used for culturing HCFs. HCFs were collected after treatments. BCA protein assay kit was used for determining the concentrations of cell lysates. 1 mg of total proteins were used for immunoprecipitation. The samples were incubated in rabbit-controlled Ig-G antibody and anti-E2F1 (1:100, Proteintech, Wuhan, China) antibody under condition of 4°C overnight. Then the samples were suspended with 25 μL of protein A/G PLUS-Agarose. After 2 h of incubation and washing, the samples were western-blotted, and anti-CCNE2 (1:1000, Abclonal, Wuhan, China) antibody was incubated to evaluate the conjunction amount of E2F1 and CCNE2.

### Statistical analysis

Datasets in bar charts are shown in the form of means ± SD. The samples were compared by non-paired student’s t-test (for 2 different groups) and One-way-ANOVA (for over 3 different groups). P value <0.05 was considered a statistically significant difference, different P values were marked over the bar chart, and all analyses and graphic drawings were performed in GraphPad Prism 7.

## Results

In our results, we found that expressions of both differentiation associated proteins and E2F1 were upregulated in TGF-β1, stimulated HCFs, silenced the expression of E2F1, demoted the expressions of differentiation associated proteins, cell proliferation, and migration abilities. We also found that CCNE2 was the downstream gene of E2F1 by luciferase assay and immunoprecipitation. E2F1 promoted the TGF-β1-induced HCFs differentiation through promoting the transcription of CCNE2.

### E2F1 was highly expressed in HCFs induced by TGF-β1

**Figure 1.**
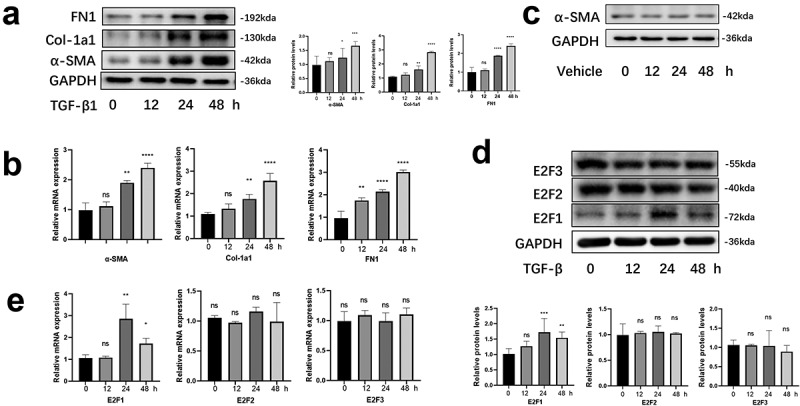
E2F1 was highly expressed in HCFs induced by TGF-β1. (a, b) Differentiation associated proteins (a) and mRNAs (b) in TGF-β1 stimulated HCFs in manner of time (loading control: GAPDH). n = 3 *p < 0.05, **p < 0.01, ***p < 0.005 ****p < 0.001 vs 0 h. C The plates that cells were cultured exerted no significant impact on differentiation. D, E Timeline of E2F1, E2F2, E2F3 protein (d) and mRNA (e) expression after TGF-β1 stimulating in manner of time. n = 3. *p < 0.05, **p < 0.01. ***p < 0.005. ****p < 0.001 vs 0 h

Western blot assay and qRT-PCR were performed to assess the fibrosis level. As a result, in TGF-β1-treated HCFs, expression of fibrosis-related genes (α-SMA, COL-1A1, FN1) was significantly increased in 12, 24 h, and reached the peak in 48 h (Figure 1(a,b)). The plates that the cells were cultured exerted no significant impact on differentiation (Figure 1(c)). Meanwhile, we observed that E2F1 expression was substantially elevated 12 h, 24 h, and reached the peak in 12 h after TGF-β1 stimulating, but the other activating transcription factor genes of E2F family (E2F2, E2F3) remain unchanged (Figure 1(d,e)). Our findings showed that E2F1 participated in fibroblast-to-myofibroblast differentiation of HCFs.

### Silenced the expression of E2F1 demoted the differentiation of HCFs

**Figure 2.**
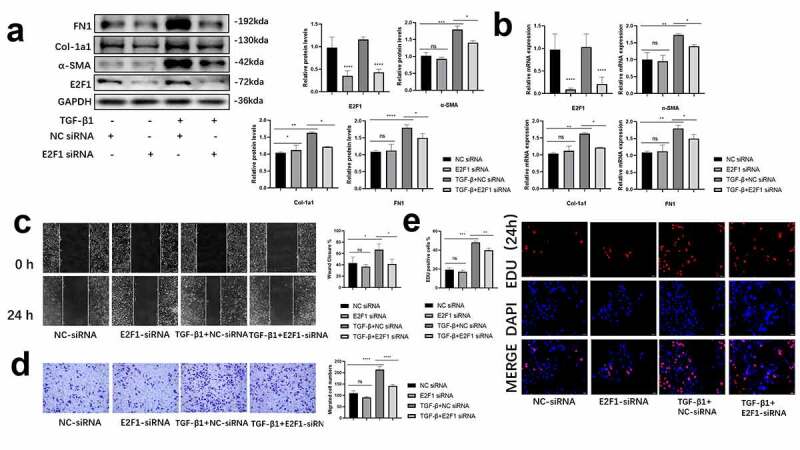
Silenced the expression of E2F1 demoted the differentiation of HCFs. (a, b) Differentiation associated proteins (a) and mRNAs (b) expression in TGF-β1 induced HCFs (loading control: GAPDH). n = 3. C-E migration (c, d) and proliferation (e) abilities of HCFs. n = 5. *p < 0.05, **p < 0.01, ***p < 0.005 ****p < 0.001

E2F1 siRNA were transfected into HCFs to further validate the effect of E2F1 on cardiac fibroblasts differentiation. Our findings showed that the mRNA and protein levels of fibrotic markers were attenuated in TGF-β1-treated HCFs with E2F1 siRNA while negative control (NC) siRNA was ineffective in non-treated HCFs (Figure 2(a,b)). Moreover, a significant inhibition in migratory ability in E2F1-silenced HCFs upon TGF-β1 stimulation was shown by the wound healing assay and transwell assay (Figure 2(c,d)). Meanwhile, cell proliferation was inhibited by E2F1 silencing in EDU assay. After TGF-β1 treatment, the number of EDU positive cells was increased but reduced significantly after E2F1 silencing (Figure 2(e)). Our findings showed that silencing the expression of E2F1 demoted the differentiation of HCFs.

### E2F1 promoted the transcription of CCNE2

**Figure 3.**
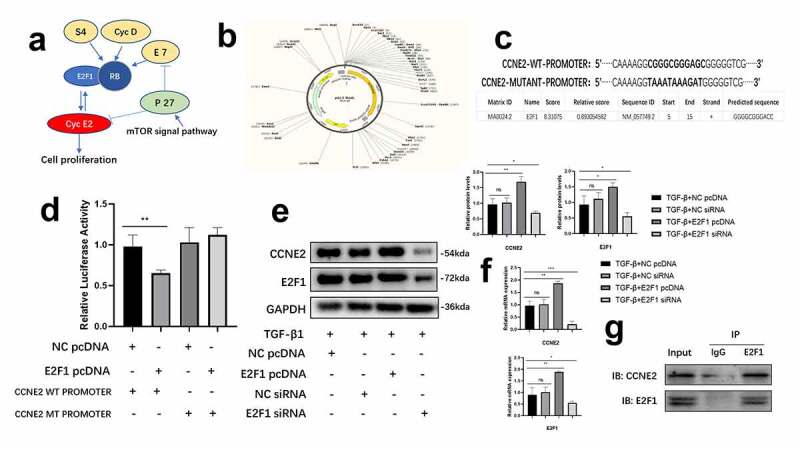
E2F1 promoted the transcription of CCNE2. (a) KEGG showed that CCNE family was key mediators in cell proliferation. (b, c) Construction of CCNE2 WT and MUTANT promoters (c) and pmirGLO vectors (b). (d) Luciferase activity in E2F1 pcDNA3.1 + CCNE2 mutant promoter. n = 4. (e, f) Expression of protein (e) and mRNA (f) of CCNE2 in TGF-β1 stimulated HCFs (loading control: GAPDH). n = 3. G Immunoprecipitation of anti-E2F1 to CCNE2 in HCFs lysates. *p < 0.05, **p < 0.01

Cellular signal pathway database KEGG [18] predicted that E2F1 promoted the gene transcription of CCEN2 (Figure 3(a)). To determine the transcriptional effect of E2F1 in CCNE2, we constructed pmirGLO vectors containing wildtype (WT) and mutant (MT) CCNE2 5ʹUTR to confirm E2F1 binding to 5ʹUTR promoter region of CCNE2 (Figure 3(b,c)). Our data showed that in WT vector, overexpression of E2F1 increased the luciferase activity, but in MT vector, no significant difference of luciferase activity was observed (Figure 3(d)). Meanwhile, we transfected E2F1 siRNA and pcDNA3.1 into TGF-β1 induced HCFs transiently, the mRNA and protein levels of CCNE2 were up-regulated in E2F1 overexpressed HCFs and down-regulated in E2F1 silenced HCFs (Figure 3(e,f)). Finally, by immunoprecipitation, we observed that E2F1 conjuncted with CCNE2 to promote the transcriptional activity of CCNE2 (Figure 3(g)). These results showed that E2F1 promoted the transcription of CCNE2.

### E2F1 promoted the TGF-β1 induced HCFs differentiation through promoting the transcription of CCNE2

**Figure 4.**
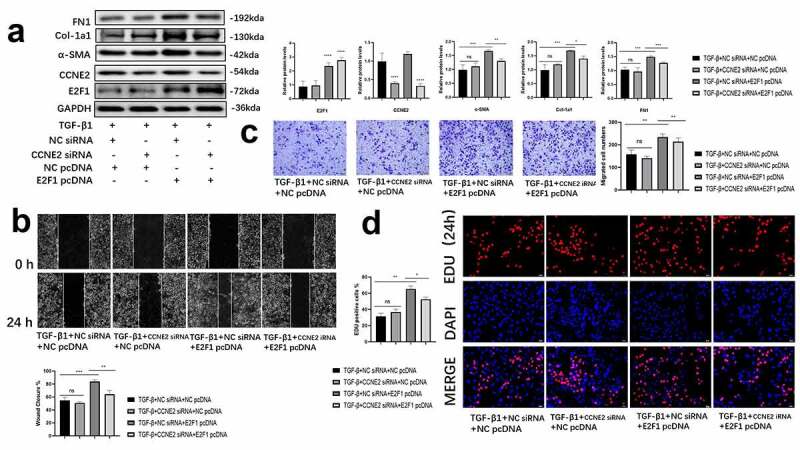
E2F1 promoted the TGF-β1 induced HCFs differentiation through promoting the transcription of CCNE2. (a) Differentiation associated proteins expression in TGF-β1 induced HCFs (loading control: GAPDH). n = 3. B-D Migratory (b, c) and proliferative (d) abilities of HCFs. n = 5. *p < 0.05, **p < 0.01, ***p < 0.005 ****p < 0.001

A siRNA was designed to silence the expression of CCNE2. We found that silenced CCNE2 significantly demoted the HCFs differentiation. When E2F1 pcDNA3.1 and CCNE2 siRNA transfected together into HCFs, CCNE2 siRNA significantly rescued the fibroblast-to-myofibroblast differentiation promoting effect of E2F1 pcDNA3.1, differentiation-associated markers were decreased in HCFs transfected with CCNE2 siRNA and E2F1 pcDNA3.1 compared with those who transfected E2F1 pcDNA3.1 alone (Figure 4(a)). Besides, the migratory and proliferative ability were also decreased (Figure 4(b–d)). The data showed above proved that E2F1 participated in the TGF-β1-induced fibroblast-to-myofibroblast differentiation of HCFs through promoting the transcription of CCNE2.

## Discussion

Excessive activation of HCFs caused by fibroblast-to-myofibroblast differentiation will lead to myocardial dysfunction and heart failure. The activation of the differentiation induced by TGF-β1 is now regarded as a critical stimulus of the process. In our research, we firstly demonstrated that E2F1 was up-regulated in TGF-β1-induced HCFs differentiation. Secondly, we silenced the expression of E2F1 and suggested that HCFs differentiation was inhibited. Then, we validated that E2F1 promoted the transcription of CCNE2. Finally, we demonstrated that the differentiation effects of overexpressed E2F1 can be rescued by CCNE2 knockdown in TGF-β1-induced HCFs. Our data proved that E2F1 promoted the TGF-β1-induced HCFs differentiation through promoting the transcription of CCNE2.

Cardiac fibrosis is a remarkable pathological process after myocardial infarction that was characterized by fibroblast-to-myofibroblast differentiation and ECM deposition [19,20], and cardiac fibrosis significantly changed the heart chamber architecture and led to heart failure at terminal stage [21]. In normal heart, cardiac fibroblasts are one of the most important cellular constituents of cardiac tissue. It plays a vital role in structural maintenance of cardiac tissue [9,22,23]. After MI, increasing the expression of TGF-β1 stimulates cardiac fibroblasts and differentiates into myofibroblasts and highly expresses differentiation-associated proteins [22]. Despite that therapeutic agents for cardiac fibrosis were already available, side effects and unknown risks have limited the application of these treatments [24]. Therefore, out findings proved that transcription factor E2F1 and its target gene CCNE2 may be a new potential target in preventing and treating of human cardiac fibrosis.

Classic cellular signal pathway of cardiac fibrosis induced by TGF-β1 is TGF-β–Smad2/3 signaling pathway. After TGF-β1 activating, it binds with its receptor TβRI and TβRII. TβRI phosphorylates its downstream transcription factor SMAD2/3. TβRI binds with SMAD4 and enters together with TβRI into cell nucleus to promote transcription of fibrosis-related genes [3,5]. Direct inhibition of TGF-β1, SMAD2/3, or TβRI/II will end up with poor outcomes like autoimmune diseases, heart failure, or unknown risks [5,22,25–27]. Hence, searching for safe and stable intervention targets for preventing and treating of human cardiac fibrosis is necessary. Transcription factors of E2F family are key regulators of numerous genes involving in DNA damage repair, cell apoptosis, and cell cycle progression. Members of E2F family are classified as either transcriptional activators or repressors. E2F1 is one of the best-studied transcription factors of E2F family, controlling cell death and cell cycle progression [28]. Research suggests that E2F1 plays a vital role in tumor invasion, proliferation, and migration [29,30]. E2F1 worked as complexes with RB, when activated. RB was phosphorylated and separated from E2F1. E2F1 got into cell nucleus to transcript downstream genes [15]. Progression between G1 and S phase is dependent on cyclin-dependent kinases (CDKs). RB and E2F1 promoted the cell proliferation and cell cycle by acting as a downstream event from the action of G1 kinases and CDKs. TGF-β1 downregulated the G1 kinase activity but upregulated CDKs activity hence converted the Rb into phosphorylated form (pRB) [31,32]. Therefore, the E2F1 was an important target of the TGF-β1 signaling pathway. Our results showed that silencing the expression of E2F1 suppressed the proliferation and migration abilities of TGF-β1-stimulated HCFs, which were consistent with reports. Recently, anti-E2F1 treatment strategy was reported by several research group [33–35], meant that E2F1 was a relatively safe and clear intervention target. Anti-E2F1 might be a better strategy for cardiac fibrosis than intervening TGF-β–Smad2/3 signaling pathway directly. But to our knowledge, no research has reported about the relationship of the E2F1 and cardiac fibrosis so far. Our data suggested that E2F1 regulated the HCFs differentiation, which meant that E2F1 participated in process of cardiac fibrosis. However, the side effect of E2F1 intervening like cytotoxicity should be validated further.

Cyclin E2 (CCNE2) is a less known member of Cyclin E family. Those highly conserved proteins regulate cell cycle by activating CDK2 to promote cell cycle progression. Cyclin E2 is less known than cyclin E1, but it is also related to proliferation. Genomic instability and high expression correlate to poor outcome in malignancies [36]. In previous reports, CCNE1 that promoting cell growth in different cell types through E2F1–cyclin E1 positive feed-back loop was well known [32], but the effect of CCNE2 in promoting the proliferation was not fully revealed until now. Some research silenced the E2F1-CCNE1-CCNE2 by siRNA and observed that the cell proliferation and migration were both inhibited [37,38], but the relationship between E2F1 and CCNE2 remains unknown. Besides, to our knowledge, the effects of CCNE2 in organ fibrosis has not been reported. As a consequence, followed by prediction, luciferase assay and immunoprecipitation results proved that CCNE2 was one of the downstream genes of E2F1, and both of them were participated in TGF-β1-induced fibroblast-to-myofibroblast differentiation of HCFs in interactions with RB, G1 kinases, and CDKs [31,32]. Like E2F1, CCNE2 was also involved in tumor invasion, proliferation, and migration [39–41]. In our research, silencing the expression of CCNE2 directly suppressed the proliferation and migration abilities of HCFs stimulated by TGF-β1, which was consistent with reports.

## Conclusion

Our study showed that E2F1 is up-regulated after TGF-β1 stimulation and E2F1 promoted TGF-β1 induced fibroblast-to-myofibroblast differentiation of HCFs by promoting the transcription of CCNE2. These findings suggested that E2F1 and CCNE2 could be a potential therapeutic target for cardiac fibrosis.

## Data Availability

The datasets supporting the conclusions of this article are included within the article and its additional files according to demands of journal. All source documents of datasets are available from the corresponding author on reasonable request.
